# Accelerated Corrosion and Multimodal Characterization of Steel Pins in High-Voltage AC Insulators Under Multi-Stress Conditions

**DOI:** 10.3390/ma18174218

**Published:** 2025-09-08

**Authors:** Cong Zhang, Heng Zhong, Zikui Shen, Hongyan Zheng, Yibo Yang, Junbin Su, Xiaotao Fu

**Affiliations:** 1 Key Laboratory of Physical and Chemical Analysis for Electric Power of Hainan Province, Haikou 570311, China; zhongheng1997@163.com (H.Z.); david830118@163.com (H.Z.); f62219726@sina.com (J.S.); fuxiaotao123abc@163.com (X.F.); 2 Electric Power Science Research Institute, Hainan Power Grid Co., Haikou 570311, China; 3 School of Electric Power Engineering, South China University of Technology, Guangzhou 510641, China; geniuszk@scut.edu.cn (Z.S.); epyyibo@mail.scut.edu.cn (Y.Y.)

**Keywords:** AC insulators, orthogonal design, salt spray, leakage current, electrochemical impedance spectroscopy, response surface modelling

## Abstract

Ensuring the long-term electro-mechanical reliability of high-voltage alternating current (HVAC) insulator strings requires a detailed understanding of how multiple environmental and electrical stressors influence the corrosion behavior of hot-dip galvanized steel fittings. In this study, a three-factor, three-level L9(3^3^) orthogonal accelerated corrosion test was conducted to systematically evaluate the individual and interactive effects of marine salt deposition (0–10 g m^−2^ day^−1^), acetic acid pollution (0–8 µg m^−3^), and 50 Hz AC leakage current (0–10 mA) on miniature pin-type assemblies. A comprehensive post-corrosion characterization approach was employed. The results revealed that chloride loading from salt deposition was the dominant contributor to corrosion. However, the synergistic interaction between salt and leakage current led to an acceleration in zinc depletion compared to the additive effect of the individual factors. A quadratic regression model with a high correlation coefficient was developed to predict corrosion volume per unit area. The findings offer a mechanistic explanation for field-reported failures in coastal power grids and provide actionable guidance for optimizing corrosion-resistant coatings and implementing electrical mitigation strategies.

## 1. Introduction

With the rapid economic development in China, particularly in coastal regions, the demand for electricity has increased significantly, driving the extensive expansion of power transmission infrastructure. Despite overall improvements in environmental conditions, metal corrosion remains a critical factor affecting the operational reliability and safety of transmission systems [1,2,3,4,5]. Corrosion can compromise the mechanical integrity of metallic components, potentially leading to structural failure or insulation breakdown. In addition, the increasing frequency of extreme weather events associated with climate change has further exacerbated corrosion risks in power grid assets [6,7]. Although corrosion in AC transmission lines has historically received less attention compared to DC systems, it is increasingly recognized as a significant threat, particularly in regions with high humidity, salt exposure, or industrial pollution. Corrosion of metal accessories—such as the steel pins and caps of porcelain insulators—can directly undermine the structural integrity of insulator assemblies and has been linked to field failures [8]. While conventional assumptions hold that AC corrosion is less severe than DC corrosion, localized corrosion induced by alternating electric fields, combined with environmental factors, has been observed to cause severe damage [8,9,10]. For example, Zhang et al. reported cases of insulator string failure in Hainan, where the most severely corroded regions were located near the cement-grouting interface—the point of maximum mechanical stress. However, the influence of environmental parameters on these failures was not further investigated [11,12].

Internationally, research on the corrosion of AC insulator metal fittings has been more active. Kim et al. [7] conducted a comprehensive analysis of steel caps and pins from porcelain insulators in service across various regions of South Korea, identifying multiple corrosion mechanisms—including galvanic corrosion, electrolytic corrosion, and crevice corrosion—depending on component type and location. They emphasized the significant role of geographic and environmental factors, proposing a pollution-level-based approach to estimate insulator service life. Sanyal et al. [13] employed Weibull distribution analysis to assess failure probabilities of 154 kV porcelain insulators at different aging stages, providing a quantitative basis for maintenance and replacement strategies. Rabelo et al. [14] introduced an improved cap design to reduce stress concentration at the steel-to-cap interface, thereby extending insulator life. Kim et al. [15] further investigated the effects of environmental pollutants—such as SO_x_, Cl^−^, and CO_2_—on steel pins and cementitious materials, highlighting carbonation-induced cracking and recommending phenolphthalein testing for pH monitoring. Sanyal et al. [16] combined experimental data with statistical modeling to evaluate the influence of temperature, pollutant concentration, and pH on corrosion rates, demonstrating a critical threshold of zinc loss (15 wt%) beyond which mechanical properties degrade significantly.

Despite these advances, limited research has systematically investigated the combined effects of multiple environmental factors—such as salt spray, acidic contamination, and AC leakage current—on the corrosion behavior of steel fittings in AC insulators, particularly under the accelerated conditions relevant to hot and humid coastal regions [17,18]. To address this gap, the present study employs an orthogonal experimental design to systematically evaluate the individual and interactive influences of these three key factors. A combination of surface characterization techniques is used to analyze corrosion morphology and the elemental composition of corrosion products. Both qualitative and quantitative assessments are conducted to elucidate the corrosion mechanisms. Furthermore, a regression model is developed to predict the volume of corrosion products, offering a practical tool for assessing corrosion severity and guiding the design of protective strategies for AC insulator steel feet in aggressive environments.

## 2. Materials and Methods

### 2.1. Orthogonal Test Design

Accelerated corrosion tests were performed in a programmable salt-spray chamber (China National Electric Apparatus Research Institute Co., Ltd, Guangzhou, China) maintained at 40 °C and 93% relative humidity, in accordance with the standard IEC 60068-2-78:2012 [19]. The salt-spray concentration was controlled by nebulizing a sodium chloride brine solution, while the level of acid pollution was simulated through periodic immersion in glacial acetic acid solutions with predetermined mass concentrations. An alternating leakage current was applied to the target area using a graphite rod held in contact with the specimen and regulated via a series resistor and a variable autotransformer (variac).

A standard L9(3^3^) orthogonal array was employed to investigate the main effects and interactions among three experimental factors: salt-spray deposition rate (0, 5, or 10 g m^−2^ day^−1^), acetic acid pollution level (0, 3, or 8 µg m^−3^), and alternating leakage current (0, 3, or 10 mA). The nine experimental runs are summarized in Table 1. For each test condition, 2 identical specimens were prepared, resulting in a total of 18 samples. The testing sequence was randomized to ensure statistical orthogonality and minimize potential bias.

### 2.2. Sample Preparation

**Figure 1:**
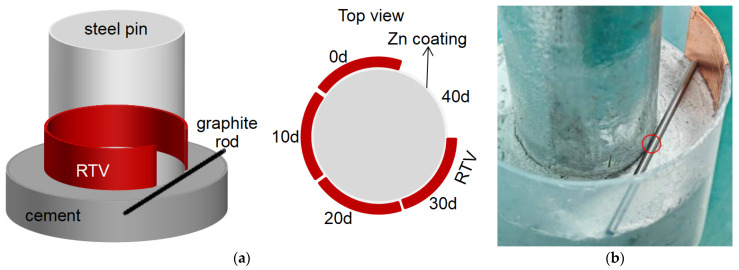
(**a**) Schematic diagram and (**b**) optical photographs of the test sample.

Figure 1a illustrates a schematic of our corrosion test samples. The steel pin with Zn coatings was embedded in concrete to replicate the field-installed configuration, and room-temperature vulcanized (RTV) silicone rubber was applied near the contact interface to control the exposure of the pin surface. Specifically, the cement-covered surface of the pin in contact with the cement matrix was divided into five equal sectors, each spanning a central angle of 72°. RTV silicone rubber was uniformly applied to four of these five sectors, with a coating thickness of approximately 1 mm, leaving one sector intentionally exposed for accelerated corrosion testing. A graphite rod with d = 2 mm was used to introduce the leakage current onto the initially exposed surface. After the initiation of the salt-spray exposure experiment, a portion of the RTV silicone rubber was removed every 10 days in a clockwise direction. As shown in Figure 1b, commercial Q235 steel pins (Ø20 mm × 60 mm) were hot-dip galvanized to achieve a zinc coating thickness of approximately 80 µm. A simulated insulator bonding structure was prepared by mixing silicate cement with water and pouring the mixture into a 250 mL polyethylene beaker. The corrosion tests were conducted in a controlled environmental chamber with a predefined testing protocol. The system operated continuously, with a scheduled pause every 10 days for half a day. During each pause, the following procedures were carried out: (1) manual removal of the RTV coating from one sector in a clockwise direction; (2) application of an acidic solution to the exposed steel surface using a brush; and (3) measurement and readjustment of the AC voltage via a variac to maintain the leakage current close to the target value. This testing approach eliminated the need for mid-test sampling, thereby reducing operational complexity. After the completion of the corrosion test, all specimens were removed from the chamber and prepared for analysis in a single step, ensuring consistency. To ensure representative results, multiple measurement points were taken from the central region of each exposed sector to minimize edge effects caused by the protective silicone coating. In addition, surface analyses were conducted along a spatial gradient from the edge of the silicone rubber toward the center, enabling evaluation of the localized protection efficiency.

### 2.3. Characterization

Following the accelerated corrosion tests, the cement-embedded steel pins were sectioned, and samples from different regions were prepared for subsequent characterization. Optical microscopy was performed using an AOSV T2 industrial stereomicroscope (Phenix Optics, Shangrao, China). Scanning electron microscopy (SEM) and energy-dispersive X-ray spectroscopy (EDS) analyses (point scan and mapping) were conducted on a Sigma 500 scanning electron microscope (Carl Zeiss AG, Oberkochen, Germany) operating at an accelerating voltage of 20 kV. Because the carbon steel surface showed minor overall corrosion and had good conductivity, no gold sputter coating was applied before testing. Optical and SEM images were captured from at least three different regions per sample. Electrochemical impedance spectra were acquired in a 0.5 mol L^−1^ Na_2_SO_4_ electrolyte at open-circuit potential using a Gamry Reference 600 potentiostat (Gary, Philadelphia, PA, USA), with a frequency range from 10 mHz to 100 kHz and an AC amplitude of 10 mV RMS. Three-dimensional surface topography was measured using coherence-scanning white-light interferometry (Up S-Dual, Rtec Instruments, San Jose, CA, USA) with a 20× objective lens [20,21] and a vertical resolution of 1 nm. The post-processing software (Gwyddion V2.63) enables the analysis of topographic parameters such as surface roughness, slope, and volume. Each sample was measured twice at different locations, with a measurement area of 0.66 mm × 0.88 mm.

## 3. Results and Discussion

To facilitate sample description, the project team specifies the abbreviation rules for sample categories as follows: the three levels of acetic acid contamination are defined as strong acid (S), weak acid (W), and no acid (N); current and salt spray are denoted by numerical values; and the number of exposure days is also represented by a numerical value. For example, “S-3-10-40” indicates a sample exposed for 40 days under test conditions of salt-spray level 10, contamination level 8, and current level 3.

### 3.1. Macroscopic Evolution of Corrosion Morphology

Table 2 presents the surface morphology of nine sample groups across different time periods. Visual inspection revealed a progressive dulling of the metallic luster. First, under fixed environmental and test parameters, it is evident that the surface condition undergoes significant changes with increasing exposure time: the metallic luster gradually disappears, the surface becomes increasingly rough, and some samples exhibit white rust or even red rust. Notably, the white rust cannot be washed off by water, ruling out salt deposition. Second, comparing the surface states of samples exposed for 40 days, the condition generally shows a positive correlation with the salt-spray level. When the salt-spray level is 10, the sample surfaces exhibit substantial white rust formation. In comparison, the influence of variations in acidic contamination is relatively minor, whereas the effect of the leakage current is more localized, with corrosion differences occurring only near the graphite electrode contact points and having negligible impact on other areas. Specifically, when both salt spray and electric current are present, raised red corrosion products appear at the contact area between the graphite electrode and the steel foot, indicating local breakthrough of the Zn barrier and cathodic depolarization of underlying Fe. These raised areas are localized near the electrical contact zone—either directly beneath the contact point or on either side of it—and are not observed in other regions. Examples include samples S-10-0-40, N-3-5-40, and N-10-10-40, as well as W-10-5-40 and S-3-10-40. However, optical images of other regions are provided in the table below (Table 2). In the experiment, graphite rods were selected as the electrodes. Since their potential is generally relatively positive, when they come into direct contact with active metals (such as zinc, magnesium, or aluminum) in an electrolyte environment, a galvanic cell is formed. However, by comparing samples W-0-10-40 with N-3-5-40 and W-10-5-40, it can be observed that when no current is applied, there are fewer corrosion products (essentially no protrusions) at the contact area. In contrast, samples subjected to current flow exhibit visible red protrusions, indicating that alternating current promotes electrochemical corrosion. Furthermore, in this experiment, the contact area between the graphite electrodes is extremely small, resulting in a current density at the contact point exceeding 100 A/m^2^. This finding is consistent with existing research [9,10], which suggests that AC current density must reach a certain threshold to accelerate corrosion.

### 3.2. SEM/EDS Microanalysis

**Figure 2:**
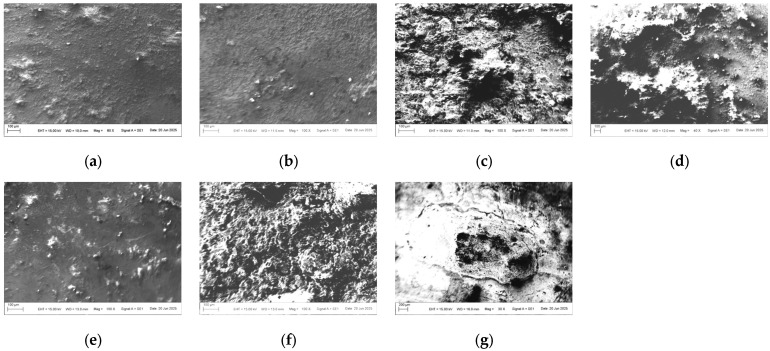
SEM image of seven representative samples: (**a**) S-0-5-40, (**b**) S-10-0-40, (**c**) S-3-10-40, (**d**) W-3-0-40, (**e**) N-0-0-0-40, (**f**) N-0-10-40, (**g**) N-3-5-40.

Based on the preliminary results of the optical images, we selected seven representative samples (from the 40-day exposure areas) to test the micro-morphology, as shown in Figure 2. Regardless of the levels of contamination and electric current, the surfaces of the samples under high-salt-spray corrosion are rough and loose, with even local cavities appearing. When the salt-spray concentration is low, most areas of the sample surfaces remain smooth, indicating that chloride ions (CI^−^) cause the greatest damage to the surface galvanized layer. Under high magnification, it can be clearly observed that the corrosion products at the electrical contact areas present mound-like shapes with missing tops (resembling volcanic craters). The missing shapes in the centers of individual corrosion product protrusions are speculated to match the graphite electrode rods. The surfaces of the product protrusions are smooth, but the interior of the missing areas is loose and porous.

**Figure 3:**
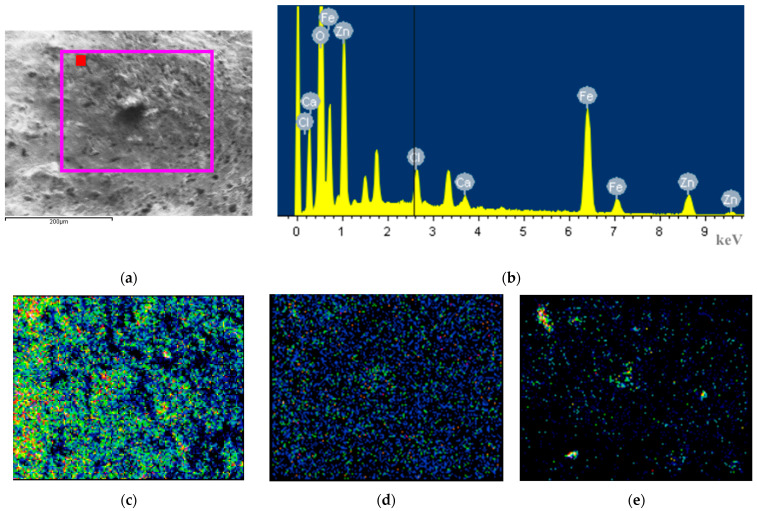
EDS results of N-3-5-40: (**a**) test region, the red dot is for point testing, the pink box is for mapping testing, (**b**) elements distribution at the test point, mapping of the (**c**) O, (**d**) Cl, (**e**) Fe.

To further determine the elemental composition of the corrosion products, energy dispersive spectroscopy (EDS) point scans and area scans were conducted in the electron microscope field of view, and the results are shown in Figure 3 and Table 3.

Judging solely from the atomic percentage of oxygen, the values follow the following order: S-10-0-40 (72.91%) ≈ W-3-0-40 (73.09%) ≈ S-3-10-40 (71.82%) > N-3-5-40 (67.35%) ≈ S-0-5-40 (65.56%) > N-0-10-40 (59.88%) ≈ N-0-0-40 (59.03%). The proportion of oxygen content can generally serve as an indicator of the degree of oxidation reaction. However, due to the non-uniformity of surface corrosion, there will still be fluctuations and errors in oxygen content. When there is no salt spray, contamination, or electric current, and the sample is only exposed to a high-temperature (35 °C) and high-humidity (90% RH) environment, the oxygen content on the surface of hot-dip galvanized coating is around 60%. After exposure to the salt-spray environment, the Cl element content on the sample surface is not high. This may be because the corrosion products dissolve in water, causing the Cl element to be washed away, or it migrates to the loose and porous interior, resulting in a relatively low surface measurement value. The relatively low Si and Ca elements may originate from cement. A small amount of Fe element is detected on the surfaces of S-0-5-40, N-0-10-40, and S-3-10-40, proving that after 40 days of exposure to a high-temperature, high-humidity, and high-salt-spray environment, the hot-dip galvanized coating is damaged. Among them, the atomic percentage of Fe on the surface of S-0-5-40 is 1.77%.

In particular, we conducted an elemental area scan on the central position of the raised corrosion products at the electrical contact area of N-3-5-40. The atomic percentage of iron at this position reaches as high as 19.1%, which is the highest among all the tested positions. At this time, the project team reasonably speculates that the injection point of alternating current will have a synergistic effect with the salt spray, especially the Cl ions, breaking through the hot-dip galvanized protection and causing deep-seated corrosion to the internal carbon steel. Since the leakage current path of alternating-current insulators has randomness and exclusivity, that is, the path depends on the distribution of soluble conductive contaminants, and once one path is conductive, the voltage borne by other paths decreases. Therefore, it is impossible for multiple paths to conduct electricity simultaneously. This situation is highly similar to the current connection method in this test. When the current enters the steel foot at a “point”, it will also have a synergistic effect with corrosive substances, resulting in weak points in the protection.

Interpreting the foregoing results through the lens of diffusion-coupled electro-chemical kinetics reveals a multifaceted corrosion scenario. Chloride ingress not only disrupts the compactness of the passive ZnO film by forming soluble zinc hydroxy-chlorides but also participates in establishing a concentrated brine that elevates the local conductivity of surface electrolytes. When an alternating leakage current is imposed, the resultant potential oscillations exacerbate film rupture, a phenomenon inadequately captured by steady-state DC polarization frameworks. Acetic pollution, often sidelined in the corrosion narrative, acts as a pH buffer that modulates the hydrolysis rates of zincate complexes, thereby exercising a second-order but statistically significant influence on both kinetics and product morphology.

### 3.3. Electrochemical Impedance Spectroscopy

To gain an in-depth understanding of the corrosion behavior and protection performance of metals in different environments from an electrochemical perspective, four samples, namely N-0-10-40, S-10-0-40, N-3-5-40, and N-10-10-40, were selected for electrochemical impedance spectroscopy (EIS) testing. These samples cover the effects of salt spray (high, low, none), acidic contamination (high, none), and leakage current (high, low, none). Wires were welded to the back of the samples, and the redundant parts were sealed with RTV, leaving only a 1 cm × 1 cm corrosion-exposed area. The samples were then immersed in a 0.5 mol/L Na_2_SO_4_ solution for testing, and the results are as follows.

**Figure 4:**
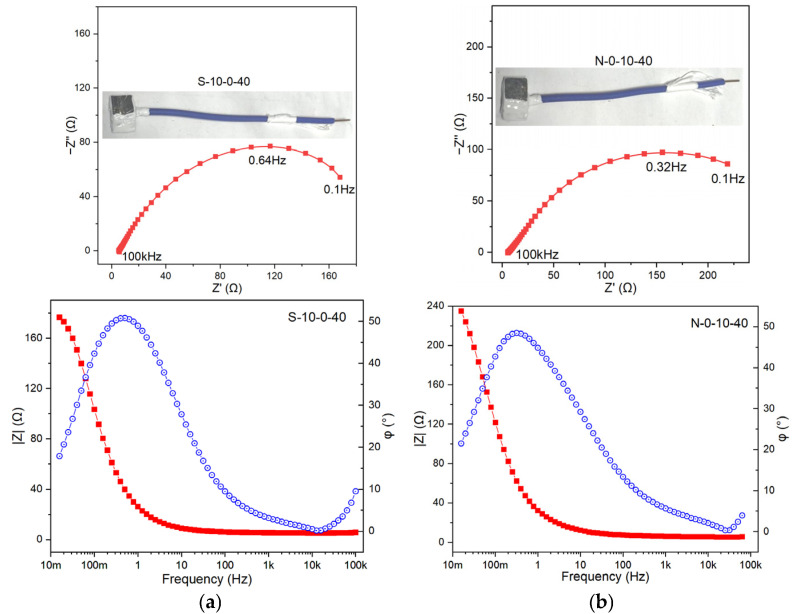

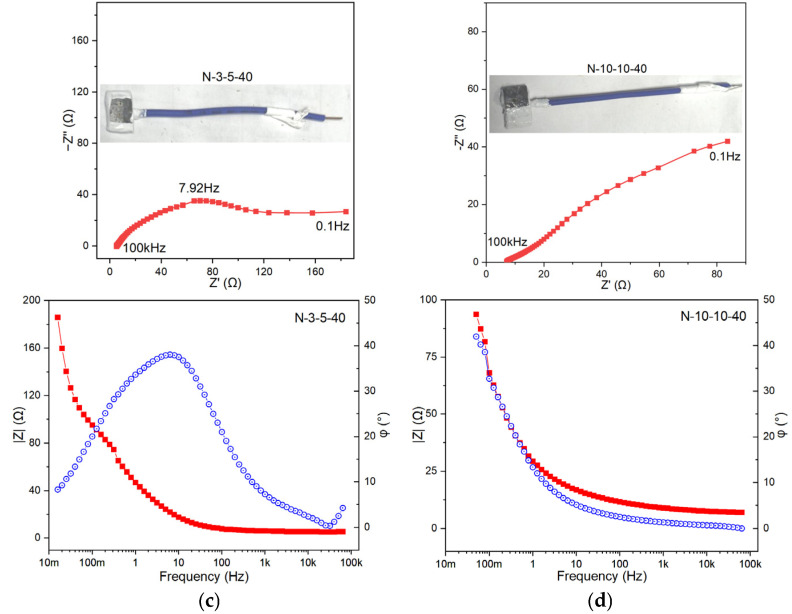
The Bode plot and frequency spectrum of electrochemical impedance. (**a**) S-10-0-40, (**b**) N-10-0-40, (**c**) N-3-5-40, (**d**) N-10-10-40.

Based on the characteristics of the Bode plots in the test results (Figure 4a–d), the |Z| (impedance modulus) of N-0-10-40 is the largest, and its phase angle reaches approximately 40°, which is a typical double-time-constant system. N-3-5-40 shows a slight inflection in the low-frequency region. The phase angle of N-10-10-40 fluctuates significantly, and the curve is unstable, suggesting a strong surface non-uniformity. The Randles equivalent circuit, the simplest and most commonly used equivalent circuit, was employed for fitting. This circuit describes the basic characteristics of the co-existence of Faraday current (charge-transfer resistance) and non-Faraday current (double-layer capacitance) at the electrode/electrolyte interface. The charge-transfer resistance (R_ct_) and double-layer capacitance (C_dl_) parameters were extracted to evaluate the corrosion behavior, interface state, and protective film performance. R_ct_ reflects the resistance of the corrosion reaction; a higher R_ct_ value indicates better corrosion resistance. C_dl_ represents the interfacial reaction activity and surface state, which are affected by factors such as surface roughness and adsorption layers. A large Cdl implies a rough surface, numerous active sites, or loose corrosion products, while a small C_dl_ indicates a dense surface or well-formed protective film. The R_ct_ values follow the order of N-0-10-40 > S-10-0-40 > N-3-5-40 > N-10-10-40. This suggests that the corrosion induced by either the electric current or the salt spray alone is limited; however, their combined effect exhibits a synergistic interaction, resulting in substantial deterioration of the protective layer. N-3-5-40 has the smallest C_dl_, suggesting a relatively dense surface or weak charge-storage capacity. N-10-0-40 and N-10-10-40 have relatively high C_dl_ values, indicating that their interfaces may be rough or have thick adsorption layers. This implies that the influence of salt spray on the metal surface is widespread, while the effect of the electric current is more localized. Notably, localized corrosion exerts a relatively small influence on C_dl_, which is consistent with the results of the surface morphology analysis.

### 3.4. 3D Profilometry and Corrosion Volume

To quantitatively investigate the volume of corrosion products per unit area, white-light interferometry mode combined with a 20× objective lens was employed. By manipulating the control handle and fine-tuning the Z-axis, continuous imaging was performed within a specific height range of targeted areas to acquire localized elevation data. Leveraging the optical system’s zoom functionality, the focal range was dynamically adjusted to ensure clear imaging of surface details (e.g., micron-scale corrosion pits) across regions with varying depths or curvatures, thereby meeting the measurement demands of complex morphologies. For larger regions of interest (e.g., those spanning welds or interfaces), the area was scanned in sections, and multiple localized 3D images were automatically stitched to generate comprehensive large-scale three-dimensional topographic data, eliminating data loss caused by field-of-view limitations during single-scan acquisitions.

**Figure 5:**
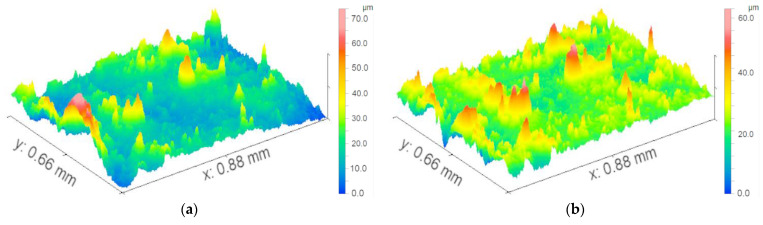
Typical results of the surfaces. (**a**) original results, (**b**) background-subtracted results.

During the post-processing phase, background subtraction was performed for curved surface features (e.g., spherical or cylindrical surfaces) on the metal samples using software. A fitting model was selected based on the actual geometric shape of the sample. Given that the sample surface was cylindrical, the least-squares method was applied to fit the cylindrical surface equation (with its axis parallel to the x-axis). The fitting parameters included the coordinates of the cylinder’s central axis and its radius. The fitting algorithm was required to achieve a residual (the deviation between raw data and the fitted surface) of <5 μm to ensure the accuracy of background subtraction. By performing point-by-point subtraction between the original 3D data and the fitted surface model, a residual surface was obtained—this surface exclusively represented the corrosion morphology (i.e., the deviation of actual corrosion pits from the ideal cylindrical surface). Typical results comparing the original and background-subtracted surfaces are presented in Figure 5. The processed images can be used to calculate morphological features such as volume, roughness and slope, as shown in Table 4.

**Figure 6:**
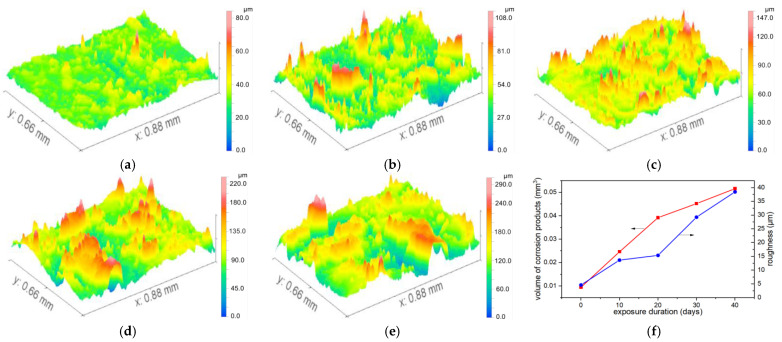
The 3D profile results of N-10-10-X are shown in (**a**–**e**), with the exposure times being 0, 10, 20, 30, and 40 days, respectively. (**f**) morphological data.

Taking the condition of salt spray = 10, pollution = 0, and current = 10 as an example, we analyzed the growth of corrosion volume and roughness within an area of 0.88 mm × 0.66 mm over exposure time, as shown in Figure 6. The results revealed that the rate of corrosion product volume accumulation began to decelerate after 20 days, indicating that excessive product buildup can inhibit further corrosion progression. The growth trends of roughness and volume are basically the same. Salt spray exhibited the largest range, identifying it as the primary factor influencing corrosion volume. Salt spray elevates electrolyte concentration, facilitating the electron-loss process of the anode metal and thereby accelerating corrosion reactions. Current ranked second, manifesting as a linear increase in the anode dissolution rate. Contamination showed the smallest range, suggesting it acts primarily as an auxiliary factor with dual effects: physical shielding and catalyzing the formation of localized micro-cells. The *p*-values for salt spray, alternating current, and contamination were all significantly <0.001, statistically confirming their strong positive impacts on corrosion volume.

**Figure 7:**
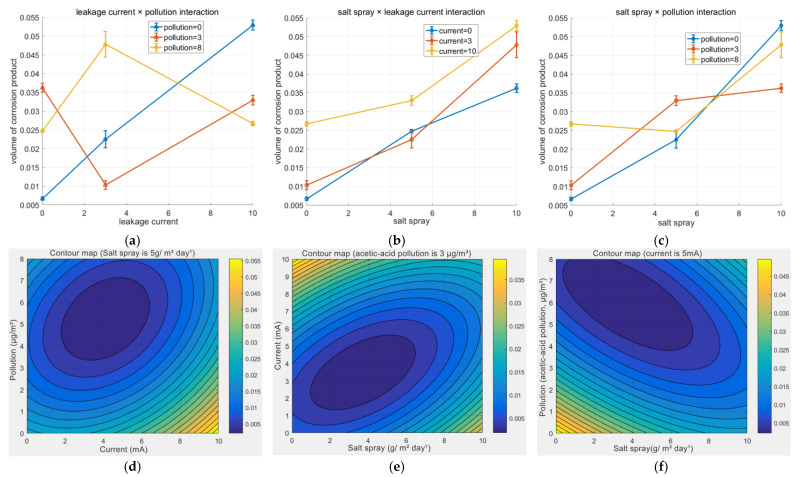
The interaction among the three factors. (**a**–**c**) line-dot plot, (**d**–**f**) contour plot.

As shown in Figure 7a–c, the interaction between salt spray and current was the most pronounced; their combined increase led to a nonlinear synergistic amplification of corrosion volume. The interaction between salt spray and pollution indicated that under high-salt-spray conditions, the barrier effect of contamination weakened. The interaction between current and pollution demonstrated that even under dominant current influence, contamination retained its regulatory role. These interaction effects collectively prove that the corrosion process is far from a simple linear superposition of individual factors. Surface roughness increased quasi-linearly with exposure time (R^2^ = 0.91) until 20 days, then plateaued. Additionally, Figure 7d–f display the corresponding contour plots, which depict the link between couple factors more clearly.

Finally, based on the result data, we used a full quadratic regression model that included main effects, quadratic terms, and interaction terms to predict the corrosion volume.

*Y* = 0.01163 + 0.0059*X*_1_ + 0.00408*X*_2_ + 0.00194*X*_3_ + 0.00015*X*_1_^2^ + 0.00012*X*_2_^2^ + 0.00003*X*_3_^2^ + 0.00012*X*_1_*X*_2_ + 0.00002*X*_1_*X*_3_ + 0.00002*X*_2_*X*_3_ (1)

Here, *Y* represents the corrosion volume, *X*_1_ stands for salt spray, *X*_2_ represents current, and *X*_3_ is pollution. Salt spray has the most significant and strongly positive impact on corrosion volume. Current is the second-most influential factor, also positively promoting corrosion. Contamination also shows a positive effect, but with a relatively small coefficient, indicating its weak independent influence. The quadratic terms of salt spray and current are negative, demonstrating the law of diminishing marginal returns (i.e., when their levels are excessively increased, corrosion no longer grows linearly). The quadratic term of contamination is nearly zero, presenting a linear trend. The positive interaction term between salt spray and current is the largest, suggesting that the combination of high salt spray and high current will synergistically amplify corrosion. As mentioned earlier, the damage caused by electric current to the metal surface primarily stems from locally high current density. Such localized high current density significantly accelerates the oxidation reaction of the metal, leading to the breakdown of the passive film and the formation of an initiation site for pitting corrosion. Subsequently, Cl^−^ ions from the environment preferentially accumulate and penetrate into this damaged area, further intensifying metal dissolution and promoting the nucleation and propagation of pitting corrosion. The interaction effects between salt spray and contamination, as well as between current and contamination, are relatively small but cannot be completely ignored.

## 4. Conclusions

This study presents a statistically robust, orthogonal experimental assessment of steel pin corrosion under the combined influence of salt spray, acidic pollution, and alternating leakage current. Among the three factors, salt spray was identified as the dominant contributor to corrosion. However, the synergistic effect between salt spray and leakage current led to the most rapid increase in material loss. The results clearly indicate that increasing zinc coating thickness alone is not sufficient to ensure long-term protection. Instead, targeted sealing of the current entry points—where corrosion is most intense-offers a more effective strategy for extending service life. The validated quadratic regression model provides a reliable tool for life prediction across a wide range of operational parameters, supporting the design of corrosion mitigation strategies in aggressive environments. These findings underscore the importance of combining metallurgical improvements-such as enhanced coatings-with electrical mitigation measures, such as leakage current density mitigation, to achieve meaningful extensions in component service life. Future work will focus on integrating finite-element electro-thermal modeling with measured field voltage gradients to further upscale the findings and enhance predictive accuracy under real-world conditions.

## Figures and Tables

**Table 1:** L9(3^3^) orthogonal experiment setup.

No.	Contamination Level (Acetic-Acid Pollution, μg/m^3^)	Leakage Current (mA)	Salt Spray (g/m^2^ Day^1^)
1	0 (N)	0	0
2	3 (W)	3	0
3	8 (S)	10	0
4	0 (N)	3	5
5	3 (W)	10	5
6	8 (S)	0	5
7	0 (N)	10	10
8	3 (W)	0	10
9	8 (S)	3	10

**Table 2:** Surface morphology of nine sample groups across different time periods.

	10d	20d	30d	40d
N-0-0	** 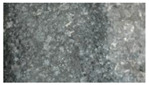 **	** 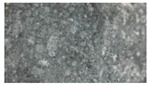 **	** 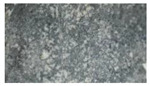 **	** 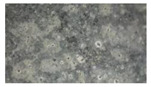 **
W-3-0	** 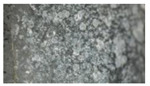 **	** 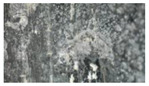 **	** 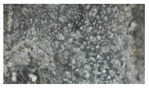 **	** 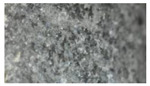 **
S-10-0	** 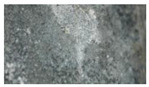 **	** 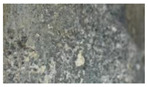 **	** 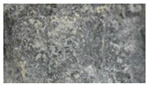 **	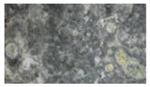
N-3-5	** 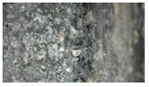 **	** 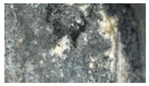 **	** 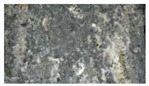 **	** 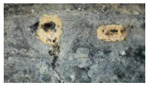 **
W-10-5	** 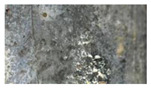 **	** 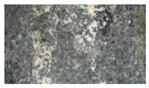 **	** 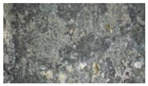 **	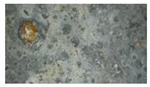
S-0-5	** 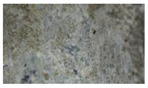 **	** 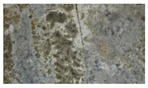 **	** 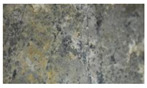 **	** 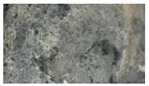 **
N-10-10	** 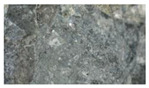 **	** 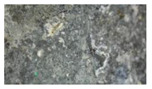 **	** 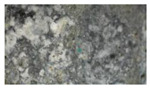 **	** 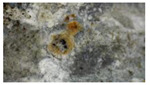 **
W-0-10	** 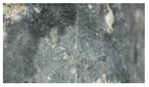 **	** 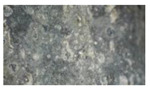 **	** 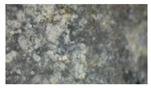 **	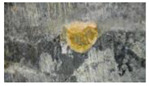
S-3-10	** 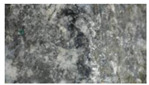 **	** 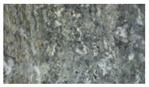 **	** 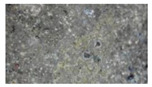 **	** 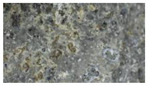 **

**Table 3:** The proportion of elements at the test point.

	Atomic Percentage						
Element	S-0-5-40	S-10-0-40	S-3-10-40	W-3-0-40	N-0-0-0-40	N-0-10-40	N-3-5-40 (Electric Contact Region)
O	65.56	72.91	71.82	73.09	59.03	59.88	67.35
Cl	1.26	0.78	0.84	0.83	0.42	0.38	2.07
Ca	10.86	5.67	2.94	3.48	1.83	1.76	0.64
Fe	1.97	0.71	1.69	1.61	0.65	0.59	19.10
Zn	21.35	19.93	22.71	20.99	38.07	37.69	10.84
Total	100.00	100.00	100.00	100.00	100.00	100.00	100.00

**Table 4:** Corrosion volume of the regions of interest after 40 days of exposure across different test groups (two sampled areas).

Samples	Salt Spray g/m^2^/day	Leakage Current mA	Pollution g/m^2^	V_1_ of Corrosion Product mm^3^	V_2_ of Corrosion Product mm^3^
N-10-10-40	10	10	0	0.05161	0.05427
W-5-10-40	5	10	3	0.03425	0.03169
S-5-0-40	5	0	8	0.02437	0.02516
S-10-3-40	10	3	8	0.0444	0.05129
S-0-10-40	0	10	8	0.02614	0.0273
W-0-3-40	0	3	3	0.0092	0.0115
W-10-0-40	10	0	3	0.0374	0.0351
N-0-0-40	0	0	0	0.0063	0.00714
N-5-3-40	5	3	0	0.0248	0.0203

## Data Availability

The original contributions presented in this study are included in the article. Further inquiries can be directed to the corresponding author.

## References

[1] Valdez B., Ramirez J., Eliezer A., Schorr M., Ramos R., Salinas R. (2016). Corrosion Assessment of Infrastructure Assets in Coastal Seas. J. Mar. Eng. Technol..

[2] Son J.-A., Choi I.-H., Koo J.-B., Kim T., Yi J. (2020). A Study on the Insulation Strength and Cement Corrosion of Porcelain Insulators in Power Transmission Line. KIEE.

[3] Leng Y. (2021). Time-Variant Probabilistic Assessment of Corrosion Initiation of Marine Concrete Structures Considering Maximum Phenomenon. Constr. Build. Mater..

[4] Lirui G., Rui Z., Fasheng H. Corrosion Factors and Protective Measures of Transmission Line Equipment in High Humidity Areas. Proceedings of the 2023 Panda Forum on Power and Energy (PandaFPE).

[5] Araújo J.A., Oliveira G.A.B., Garcia M.A., Mendonça N.M., Da Silva C.R.M., Garcia P.A.N., Badibanga R.K. (2023). A Case Study of Glass Insulator Pin Failure in 500 kV Transmission Line. Eng. Fail. Anal..

[6] Wang C., Chowdhury S., Liang X. (2024). Transmission Structure Corrosion Due to Stray Currents and the Inspection and Mitigation Techniques: A Review. IEEE Trans. Ind. Appl..

[7] Kim T., Lee Y.-J., Sanyal S., Woo J.-W., Choi I.-H., Yi J. (2020). Mechanism of Corrosion in Porcelain Insulators and Its Effect on the Lifetime. Appl. Sci..

[8] Thakur A.K., Arya A.K., Sharma P. (2020). The Science of Alternating Current-Induced Corrosion: A Review of Literature on Pipeline Corrosion Induced Due to High-Voltage Alternating Current Transmission Pipelines. Corros. Rev..

[9] Liang Y., Du Y. (2020). Research Progress on Evaluation Criteria and Mechanism of Corrosion Under Cathodic Protection and AC Interference. J. Chin. Soc. Corros. Prot..

[10] Li C., Chen X., He C., Li H., Pan X. (2021). Alternating Current Induced Corrosion of Buried Metal Pipeline: A Review. J. Chin. Soc. Corros. Prot..

[11] Nishikata A., Zhu Q., Tada E. (2014). Long-Term Monitoring of Atmospheric Corrosion at Weathering Steel Bridges by an Electrochemical Impedance Method. Corros. Sci..

[12] Kumar C.M.P., Chandrashekarappa M.P.G., Kulkarni R.M., Pimenov D.Y., Giasin K. (2021). The Effect of Zn and Zn–WO3 Composites Nano-Coatings Deposition on Hardness and Corrosion Resistance in Steel Substrate. Materials.

[13] Sanyal S., Kim T., Seok C.-S., Yi J., Koo J.-B., Son J.-A., Choi I.-H. (2020). Replacement Strategy of Insulators Established by Probability of Failure. Energies.

[14] Rabelo M., Kim T., Sanyal S., Kim K., Seok C.-S., Choi I.-H., Yi J. (2022). The Impact of Cap Orientation on Mechanical Strength of High Voltage Devices and a Novel Design for Improvement. J. Braz. Soc. Mech. Sci. Eng..

[15] Kim T., Sanyal S., Rabelo M., Yi J. (2022). Analysis of the Deterioration of High-Voltage Insulators in Service Areas Due to Contamination Factors. ECS J. Solid State Sci. Technol..

[16] Sanyal S., Kim T., Rabelo M., Pham D.P., Yi J. (2023). Experimental and Statistical Approach to Detect the Corrosion Rate and Influencing Profiles for Enhancing Corrosion Rate of High-Voltage Insulator Materials. Appl. Biochem. Biotechnol..

[17] Rachmawati R., Sartika N., Meisa Putra N.R., Suwarno S. (2018). The Study on Leakage Current Characteristics and Electrical Properties of Uncoated Ceramic, RTV Silicon Rubber Coated Ceramic, and Semiconducting Glazed Outdoor Insulators. IJEEI.

[18] Rahim N.A.A., Ranom R., Zainuddin H., Razak I.A.W.A. (2019). Numerical Simulation of Leakage Current on Conductive Insulator Surface. IJRTE.

[19] (2012). Environmental Testing-Part 2-78: Tests-Test Cab: Damp Heat, Steady State.

[20] Kushwaha P., Soni S. Corrosion Detection by Image Processing. https://www.academia.edu/download/63612769/IJSRED-V3I3P6820200612-87926-1ak9oxw.pdf.

[21] Homborg A., Mol A., Tinga T. (2024). Corrosion Classification through Deep Learning of Electrochemical Noise Time-Frequency Transient Information. Eng. Appl. Artif. Intell..

